# The effect of hospital-based health promotion on the health practices of full-time hospital nurses: a cross-sectional study

**DOI:** 10.1038/s41598-023-36873-z

**Published:** 2023-06-16

**Authors:** Hung-Hui Chen, Jerry Cheng-Yen Lai, Shu-Ti Chiou, Nicole Huang, Li-Yin Chien

**Affiliations:** 1 School of Nursing, College of Medicine, National Taiwan University, Taipei, Taiwan, ROC; 2 Department of Nursing, National Taiwan University Hospital, Taipei, Taiwan, ROC; 3 Department of Medical Research, Taitung MacKay Memorial Hospital, Taitung, Taiwan, ROC; 4 Master Program in Biomedicine, College of Science and Engineering, National Taitung University, Taitung City, Taiwan, ROC; 5 College of Medicine, National Yang Ming Chiao Tung University, Taipei, Taiwan, ROC; 6 Cheng Hsin General Hospital, Taipei, Taiwan, ROC; 7 Institute of Hospital and Health Care Administration, National Yang Ming Chiao Tung University, Taipei, Taiwan, ROC; 8 Institute of Community Health Care, College of Nursing, National Yang Ming Chiao Tung University, Yang-Ming Campus, No.155, Sec. 2, Linong St., Beitou Dist., Taipei City, 11221 Taiwan, ROC

**Keywords:** Health care, Health occupations

## Abstract

Many studies have reported positive contributions of health promotion on the health behavior of nursing staff working in hospitals, including the maintenance of a regular healthy diet, engagement in physical activity, performance of routine screening practices, and participation in a health examination. Despite being considered a role model for healthy lifestyles, little is known about the effect of health-promoting hospital settings on nursing staff. The aim of this study was to perform a nationwide, hospital-based, cross-sectional, survey comparing health practices between full-time nurses of health-promoting hospitals and those of non-health-promoting hospitals in Taiwan. We conducted a nationwide, hospital-based, cross-sectional, survey in 100 hospitals from May to July 2011 using a questionnaire as the measurement tool. Nurses aged between 18 and 65 years from certified health-promoting hospitals (n = 14,769) were compared with nurses in non-health-promoting hospitals (n = 11,242). A multiple logistic regression model was conducted to estimate the effect of certified HPH status on the likelihood of performing health behavior, receiving general physical examination, undergoing cancer screening, and participating in hospital-based health-promoting activities. All nurses of HPH hospitals were more likely to perform physical activity, practice cancer screening, receive at least one general physical examination in the past 3 years, and had a higher chance of participating in at least one hospital-based health-promoting activity in the past year (particularly weight-control groups and sports-related clubs) than those of non-HPH hospitals. This study suggests the effectiveness of implementing health promotion on the health behavior of full-time nursing staff in hospitals.

## Introduction

Health promotion and disease prevention are integral parts of primary healthcare management. Ever since the mid-1980s, the Ottawa Charter for Health Promotion has received considerable attention globally^1^. In 1988, the World Health Organization (WHO) initiated the Network of the health-promoting hospital (HPH). The HPH initiative aims at reorienting hospital services and resources toward health promotion and disease prevention^2^. The implementation of HPH has been recognized as a core strategy to encourage healthier lifestyles and behaviors in disease prevention among healthcare workers and patients. Many studies have reported the positive contribution of health promotion on the health behavior of nursing staff working in hospitals, including the maintenance of a regular healthy diet, engagement in physical activity, performance of routine screening practices, and participation in health examinations^3–7^. Since its establishment, the HPH initiative has now been adopted by more than 700 hospitals and health service members in more than 40 countries worldwide. The Taiwan HPH Network became the first Asian member of the WHO's international HPH network in 2006. With strong commitment from the Taiwanese health authorities, it has become the largest domestic HPH network globally with 162 members by 2016 (147 hospitals, 13 township public health centers, and 2 long-term care facilities)^2^.

Despite the fact that physicians have ultimate responsibility for the care of their patients, hospital authorities continue to increase the work responsibility of nurses to implement patient care^8^. Being the largest primary health care professional group working at hospitals, the health practice of nurses is important because they spend more time with their patients, and provide more direct care with them than the doctors. Nurses who practice healthy behaviors may influence their patients to adopt healthy lifestyles^9–13^. In fact, the general public has more confidence in nurses who are normal-weight to provide general advice about diet and such as reducing calorie intake and increasing exercise to overweight or obese patients to achieve weight loss than those who are overweight^13,14^. Nurses who are ex-smokers or non-smokers have more positive attitudes and are more motivated to engage in smoking cessation for their patients than nurses who smoked^11,12^. When compared to nurses who are physically inactive, nurses who regularly exercise are more likely to encourage physical activity among patients^9,10^.

The HPH initiative also had a positive impact on implementing health promotion strategies among hospitals in Taiwan^3,15–19^. Although HPH had a positive effect on hospital workers, the nursing staff had significantly fewer days “exceeding 30-min of walking or equivalent physical activity” and “having 5 portions of fruits and vegetables” during the past week as well as were less likely to participate in health-promoting activities provided by hospitals (participation in sports-related clubs, weight-control groups, and recreational or service clubs; use of gym or sports equipment; attending lectures) than physicians, pharmacists, and other health professionals^3^. Notwithstanding their increased awareness and greater accessibility to healthcare services, nurses exhibit lower rates of Pap smear screening in comparison to the general population^6^. Variances in individual characteristics, encompassing age, gender, and comorbidity, may significantly influence an individual's health-promoting practices. A previous study has identified that male healthcare professionals or healthcare professionals aged over 45 years have a higher likelihood of engaging in physical activity^19^. Nevertheless, little is known about the effect of health-promoting hospital settings on the overall health practices (health behavior, health screening, and participation in hospital-based health-promoting activities provided by hospitals) of nursing staff alone, which is particularly important when considering them as a role model for healthy lifestyles. Moreover, little is known about the interrelationship among health-promoting hospital settings, individual differences (age, gender, and comorbidity), and health-promoting practices in nurses.

Health-promoting hospital settings and individual differences (age, gender, and comorbidity) are independent and significant predictors of health practices among healthcare professionals. In addition, age, gender, and comorbidity of individuals were considered to affect the choices of lifestyle, which may change the effect of health-promoting hospital settings on health practices. This study examined the relationship between health-promoting hospital settings and health practices among nurses using a nationwide, hospital-based survey in Taiwan. We further examined whether this relationship was moderated by age, gender, and baseline chronic disease.

## Subjects and methods

### Study design and participants

In Taiwan, all Taiwan’s civilian residents were covered under a single-payer government-operated insurance program. A global budget was negotiated on an annual basis between the Department of Health and healthcare providers of all accredited hospital levels (district hospital, regional hospital, or medical center). Private providers dominate Taiwan’s healthcare market.

We conducted a nationwide, hospital-based, cross-sectional, survey in 100 hospitals from May to July 2011 using the questionnaire as a measurement tool. The questionnaire was developed and collected by the Taiwan Bureau of Health Promotion (BHP) to assess the personal health practices, health-related behaviors, and psychosocial work environment of full-time hospital staff members. Informed consent was obtained from all participants. To protect survey respondents’ confidentiality, data were collected using a self-administered, anonymous, and structured questionnaire, which was developed for comprehension and ease of completion among hospital workers. Cover letters, paper-based questionnaires, and reply envelopes were sent to 98,817 full-time staff members at the participating hospitals. After completing the questionnaire, participants were asked to place the anonymous questionnaire in a sealed envelope and return it to a collection site at the hospital. The study protocol was approved by the Institutional Review Board of the Taiwan BHP (Investigation No. 0990800708). The study was performed in accordance with the Declaration of Helsinki. The details of the study design are described in our previous investigation^20^.

We referred certified HPH hospitals to member hospitals of the Taiwan HPH Network. In 2011, there were 66 certified HPH (6 medical centers, 45 regional hospitals, and 15 district hospitals) and 421 non-HPH certified hospitals (17 medical centers, 40 regional hospitals, and 364 district hospitals) in Taiwan. To ensure a fair representation, we randomly selected non-Health Promoting Hospitals using a 1:1 ratio based on the distribution of accredited hospital levels. According to the accredited hospital level, we invited all 66 certified HPH and 61 randomly matched non-HPH certified hospitals to participate in the study. A total of 100 hospitals [55 (83.3%) certified HPH and 45 non-HPH certified (81.8%) hospitals] agreed to participate in this survey.

We received 70,622 completed questionnaires, for a response rate of 71.5% (73.6% from HPH and 68.7% from non-HPH certified hospitals). A total of 33,592 respondents reported themselves as full-time licensed registered or practical nurses. To maintain homogeneity in work patterns as suggested by a previous study^19^, we only included nurses who worked in the five main hospital units, including the operating or delivery room; outpatient clinic; emergency room or intensive care unit; general ward; or administration department. Since the mandatory retirement age for insured workers was 65 in Taiwan, we excluded respondents aged less than 18 and older than 65 years. After excluding those with incomplete information or missing information on independent variables of interest, a total of 26,011 nurses aged between 18 and 65 years were included in the study cohort. Nurses from the certified HPH hospitals were included in the HPH group (n = 14,769, 56.8%), and the remaining nurses in the non-HPH group (n = 11,242, 43.2%; see Supplementary Fig. S1).

### Measurements

Independent covariates included baseline participant characteristics (age, sex, education level, marital status), health status (obese status, presence or absence of chronic disease), healthy lifestyle (smoking and drinking status), work unit, and hospital characteristics (accredited level and ownership). *Age* was assessed in years and subsequently divided into two categories: 18–39 years and 40–65 years. *Educational level* options encompassed junior high school or below, senior high school, vocational school, university, and post-graduate education. *Marital status* was determined as unmarried, married, separated, divorced, or widowed. The body-mass index cut-off point for *obesity* (≥ 27 kg/m^2^) was defined by the Health Promotion Administration in Taiwan. *Chronic diseases* included but were not limited to diabetes, hypertension, hyperlipidemia, viral hepatitis, insomnia, asthma, and fatty liver. *Smoking status* was stratified into three categories: never, former, and current; while *drinking habits* were categorized into never, occasional, small regular amounts, and large regular amounts. *Accredited hospital level* (medical center, regional hospital, or district hospital) was stipulated by the Taiwan Joint Commission on Hospital Accreditation.

The primary outcomes of the study included (1) health behavior (physical activity and dietary behavior); (2) health screening (general physical examination and cancer screening practice), and (3) participation in hospital-based health-promoting activities (attending lectures, participation in sports-related clubs, use of gym or sports equipment, participation in weight-control groups, and participation in recreational or service clubs).

Health behaviors (*physical activity* and *dietary behavior*) were determined by enquiring the number of days "walking exceeding 30 or more minutes or equivalent physical activity" and "eating at least five portions of fruits and vegetables" during the past week. The frequency of physical activity and dietary behaviors was divided into 0, 1–2, 3–4, 5–6, and 7 days per week.

Health screening (general physical examination and cancer screening practices) was enquired as "how long it had since their last examination (including general physical examination, Papanicolaou (Pap) smear, mammography examination, and fecal occult blood test)". *General physical examination* was measured using a 5-point Likert-scale item from 1 (none), 2 (more than 6 years), 3 (4–6 years), 4 (1–3 years), and 5 (less than one year). A *Pap smear* (also known as Pap test) is a screening method used to detect cervical cancer in women. The time elapsed since the last pap smear test was divided into > 6 years, 4–6 years, 1–3 years, < 1 year, never and females older than 30 years, never and females younger than 30 years, and male (not applicable). *Mammography examination* was measured using a 6-point Likert-scale item from 1 (less than 2 years), 2 (2–4 years), 3 (more than 4 years), 4 (never and female older than 40 years), 5 (never and female younger than 40 years), to 6 (male). *Fecal occult blood test* was measured using a 4-point Likert scale item from 1 (never), 2 (more than 4 years), 3 (2–4 years), to 4 (less than 4 years).

Participation in hospital-based health-promoting activities on a healthy diet and sport-related fitness was determined by enquiring "during the past year, did you participate in the indicated activities (including lectures, clubs/groups, and use of gym or sports equipment)?" *Participation in health-related lectures* was measured using a 3-point Likert scale item from 1 (none), 2 (a couple of times) to 3 (often), whereas *participation in clubs/groups* and *use of gym or sports equipment* were measured using a 5-point Likert scale item from 1 (none), 2 (less than once time a month), 3 (at least once a month), 4 (once or twice a week) to 5 (more than 3 times a week).

We constructed our primary outcomes as binary variables into none or at least one incidence (including at least one day walking exceeding 30 min or five portions of fruits and vegetables during the past week; at least one general physical examination in the past 3 years, Pap smear in the past 3 years (all age or older than 30 years), mammography examination in the past 2 years (all age or older than 45 years), fecal occult blood test in the past 2 years; and participated in at least one hospital-based health-promoting activities in the past year).

### Statistical analysis

The dissimilarities in baseline participant and hospital characteristics, health status, and work unit between the HPH and non-HPH hospitals were compared using the Student’s *t-*test for continuous variables and Pearson’s chi-square test (χ^2^) for categorical variables. The multivariate logistic regression model was conducted to estimate the effect of certified HPH status on the likelihood of performing health behavior, receiving general physical examination, undergoing cancer screening, and participating in hospital-based health-promoting activities, with adjustment for participant and hospital characteristics, health status, health behavior, and work unit. To account for the potential interaction effects between certified HPH status and individual differences on health practices, sex (male or female), age (younger or older than 40 years), and chronic disease were also used as stratification variables in the logistic model. All data transformation and statistical analyses were performed using SAS 9.4 statistical software (SAS Institute Inc., Cary, NC, USA). All statistical assessments were considered significant at *P* < 0.05 based on two-sided tests.

## Results

### Baseline characteristics

Table 1 compares the baseline participant characteristics, health behavior, work unit, and hospital characteristics between nurses in the HPH and non-HPH hospitals. Slightly more than half of the nurses worked in the HPH hospitals (56.8%). Most nurses were women (98.2%), aged between 18 and 39 years (84.8%), holders of a university or postgraduate degree (57.7%), unmarried (56.2%), and workers in private (67.5%) or regional hospitals (65.3%). Only 10.1% of nurses were obese and about 27.3% reported having chronic diseases, particularly insomnia (14.4%), fatty liver (4.7%), and lipidemia (4.6%). Less than 1.3% of nurses were current smokers and 2.6% were regular drinkers. Overall, most nurses worked in the general ward 11,452 (44.0%), followed by 6608 (25.4%) in the emergency room or ICU, 4062 (15.6%) in the outpatient clinic, 3226 (12.4%) in operating or delivery room, and 663 (2.5%) in administration.

**Table 1:** Baseline participant characteristics, health behavior, work unit, and hospital characteristics of nurses by the health-promoting certification status of hospital (N = 26,011).

Independent variable	HPH group	Non-HPH group	*p* value^a^
	(n = 14,769; 56.8%)	(n = 11,242; 43.2%)	
Participant characteristics			
Mean age (SD), y	32.1 (7.5)	32.1 (7.0)	0.781
Age group, n (%)			
18–39 y	12,427 (84.1)	9621 (85.6)	0.001
40–65 y	2342 (15.9)	1621 (14.4)	
Sex (male), n (%)	238 (1.6)	223 (2.0)	0.024
Education level, n (%)			
High school or lower	367 (2.5)	276 (2.5)	< 0.001
Vocational school	6236 (42.2)	4129 (36.7)	
University	7824 (53.0)	6535 (58.1)	
Post-graduate	342 (2.3)	302 (2.7)	
Marital status, n (%)			
Unmarried	8436 (57.1)	6194 (55.1)	0.002
Married	5984 (40.5)	4786 (42.6)	
Separated	69 (0.5)	35 (0.3)	
Divorced	237 (1.6)	202 (1.8)	
Widowed	43 (0.3)	25 (0.2)	
Health status			
Obese status, n (%)	1528 (10.3)	1100 (9.8)	0.137
Chronic disease, n (%)			
Any chronic disease	4098 (27.7)	2992 (26.6)	0.042
Diabetes	148 (1.0)	102 (0.9)	0.438
High blood pressure	457 (3.1)	307 (2.7)	0.085
Lipidemia	708 (4.8)	481 (4.3)	0.049
Viral hepatitis	534 (3.6)	439 (3.9)	0.223
Insomnia	2135 (14.5)	1605 (14.3)	0.683
Asthma	471 (3.2)	317 (2.8)	0.085
Fatty liver	706 (4.8)	527 (4.7)	0.728
Health behavior			
Smoking, n (%)			
Never	14,204 (96.2)	10,885 (96.8)	0.018
Quit	369 (2.5)	229 (2.0)	
Current	196 (1.3)	128 (1.1)	
Drinking, n (%)			
Never	9497 (64.3)	7374 (65.6)	0.064
Occasionally	4862 (32.9)	3593 (32.0)	
Small quantity regularly	367 (2.5)	253 (2.3)	
Large quantity regularly	43 (0.3)	22 (0.2)	
Unit, n (%)			
Administration	394 (2.7)	269 (2.4)	0.493
General ward	6482 (43.9)	4970 (44.2)	
Emergency room or ICU	3787 (25.6)	2821 (25.1)	
Outpatient clinic	2292 (15.5)	1770 (15.7)	
Operating or delivery room	1814 (12.3)	1412 (12.6)	
Hospital characteristics			
Accredited hospital level, n (%)			
District hospital	1437 (9.7)	630 (5.6)	< 0.001
Regional hospital	9988 (67.6)	7009 (62.3)	
Medical Center	3344 (22.6)	3603 (32.0)	
Hospital ownership (Private), n (%)	9461 (64.1)	8091 (72.0)	< 0.001

*HPH* hospital promoting hospital, *ICU* intensive care unit.

^a^Unadjusted *p*-value (χ^2^ test or t test).

### Unadjusted and adjusted analyses by certified HPH status of the hospital

Table 2 compares the adjusted and unadjusted odds ratio of health-related behaviors and screening practices associated with the HPH status of the hospital. The dietary intake of five portions of fruit and vegetables in at least one day during the past week was high for all nurses (85.5%-86.3%). Nurses of HPH hospitals were more likely to perform physical activity (adjusted odds ratio [aOR] 1.12; 95% confidence interval [CI], 1.07–1.19) or receive at least one general physical examination in the past 3 years (aOR 1.27; 95% CI, 1.18–1.36) than those of non-HPH hospitals. When considering cancer screening practice alone, a significantly higher proportion of nurses in HPH hospitals practiced cancer screening than those in the non-HPH hospitals (55.1% vs 47.0%, *P* < 0.001). After adjusting for participant and hospital characteristics, health status, health behavior, and work unit, only physical activity, the HPH effect increased the cancer screening practices by 62% (aOR 1.62; 95% CI 1.53–1.71), particularly the increased in fecal occult blood test screening in the past 2 years by 153% (aOR 2.53; 95% CI 2.37–2.70). Overall, the participation level in at least one hospital-based health-promoting activity in the past year was high for all nurses (43.0–46.0%). All nurses of HPH hospitals had a higher chance of participating in most activities, particularly sports-related clubs (24.7% vs 20.7%, *P* < 0.001) and weight-control groups (13.4% vs 8.9%, *P* < 0.001). However, there were no significant differences in attending health-promoting lectures between nurses of HPH and non-HPH hospitals even in stratified analyses by sex, age, and chronic disease.

**Table 2:** Unadjusted and adjusted odds ratio of health-related behaviors and screening practices associated with the health-promoting certification status of hospital (N = 26,011).

Outcomes	HPH group	Non-HPH group				
	(n = 14,769) (56.8%) n (%)	(n = 11,242) (43.2%) n (%)	OR (95%CI)	*p-*value^a^	aOR (95%CI)^b^	*p-*value
Health behavior (at least one day during the past week)						
Exceeding 30 min of walking or equivalent physical activity	9945 (67.3)	7240 (64.4)	1.14 (1.08–1.20)	< 0.001	1.12 (1.07–1.19)	< 0.001
Five portions of fruits and vegetables	12,752 (86.3)	9611 (85.5)	1.07 (1.00–1.15)	0.050	1.08 (1.00–1.16)	0.043
Health screening						
General physical examination in past 3 y, n (%)	12,725 (86.2)	9266 (82.4)	1.33 (1.24–1.42)	< 0.001	1.27 (1.18–1.36)	< 0.001
Cancer screening practice						
Any cancer screening practice^c^	8138 (55.1)	5280 (47.0)	1.39 (1.32–1.46)	< 0.001	1.62 (1.53–1.71)	< 0.001
Pap smear in past 3 y (female nurses only)	5777 (39.8)^e^	4153 (37.7)^e^	1.10 (1.04–1.15)	< 0.001	1.22 (1.15–1.31)	< 0.001
Pap smear in past 3 y (female nurses older than 30 y)	4693 (57.2)^f^	3464 (53.3)^f^	1.17 (1.10–1.25)	< 0.001	1.28 (1.18–1.39)	< 0.001
Mammography in the past 2 y (female nurses only)	1480 (10.2)^e^	851 (7.7)^e^	1.36 (1.25–1.49)	< 0.001	1.38 (1.26–1.52)	< 0.001
Mammography in the past 2 y (female nurses older than 45 y)	548 (47.5)^g^	250 (33.7)^g^	1.78 (1.47–2.15)	< 0.001	1.90 (1.55–2.31)	< 0.001
Fecal occult blood test in past 2 y	4107 (27.8)	1625 (14.5)	2.28 (2.14–2.43)	< 0.001	2.53 (2.37–2.70)	< 0.001
Health-promoting activity in the past year						
Any health-promoting activity	6787 (46.0)	4838 (43.0)	1.13 (1.07–1.18)	< 0.001	1.15 (1.09–1.21)	< 0.001
Attend lectures	3421 (23.2)	2556 (22.7)	1.02 (0.97–1.09)	0.417	1.03 (0.97–1.09)	0.398
Participation in sports-related clubs	3650 (24.7)	2327 (20.7)	1.26 (1.19–1.33)	< 0.001	1.33 (1.25–1.41)	< 0.001
Use of gym or sport equipment	1253 (8.5)	847 (7.5)	1.14 (1.04–1.25)	0.005	1.16 (1.06–1.27)	0.002
Participation in weight-control groups	1976 (13.4)	996 (8.9)	1.59 (1.47–1.72)	< 0.001	1.53 (1.41–1.66)	< 0.001
Participation in recreational or service clubs	1228 (8.3)	857 (7.6)	1.10 (1.00–1.20)	0.042	1.13 (1.03–1.24)	0.012

*aOR* adjusted Odds Ratio, *CI* confidence interval, *HPH* health-promoting hospital, *y* years.

^a^Unadjusted *p*-value (χ^2^ test).

^b^Odds ratio adjusted for participant characteristics (age, sex, education level, marital status), health status (obese status, presence or absence of chronic disease), health behavior (smoking and drinking status), work unit, and hospital characteristics (accreditation level and ownership).

^c^Any cancer screening practice included pap smear test in the past 3 years, mammography in the past 2 years, and fecal occult blood test in the past 2 years in female nurses, but refereed to only fecal blood test in the past 2 years in male nurses.

^d^Odds ratio adjusted for all variables listed in footnote b, except for the sex status.

^e^Female [Non-HPH (n = 11,019; 43.1%)/HPH groups (n = 14,531; 56.9%)].

^f^Female [Non-HPH (n = 6499; 44.2%)/HPH groups (n = 8206; 55.8%)].

^g^Female [Non-HPH (n = 741; 39.1%)/HPH groups (n = 1154; 60.9%)].

^h^Odds ratio adjusted for all variables listed in footnote b, except for the sex and age status.

The associations of HPH status, sex, age, and chronic disease with health practices are presented in Tables 3, 4, and 5. The interactions between HPH status and sex, between HPH status and age as well as between HPH status and chronic disease were significantly associated with health practices. Male nurses in HPH (aOR 2.53, 95% CI: 1.81–3.54), male nurses in non-HPH (aOR 2.12, 95% CI: 1.52–2.94), and female nurses in HPH (aOR 1.12, 1.07–1.18) were more likely to engage in exceeding 30 min of walking or equivalent physical activities when compared to female nurses in non-HPH settings. Male nurses in HPH, male nurses in non-HPH, and female nurses in HPH were also more likely to participate in sports-related clubs and use gym or sport equipment provided by hospitals when compared to female nurses in non-HPH settings.

**Table 3:** Adjusted odds ratio of health-related behaviors and screening practices associated with the interaction effect of health-promoting certification status of hospital and sex (reference: female and non-HPH group) (N = 26,011).

Outcomes	Female and HPH group aOR (95%CI)^a^	*p-*value	Male and Non-HPH group aOR (95%CI)^a^	*p-*value	Male and HPH group aOR (95%CI)^a^	*p-*value
Health behavior (at least one day during the past week)						
Exceeding 30 min of walking or equivalent physical activity	1.12 (1.07–1.18)	< 0.001	2.12 (1.52–2.94)	< 0.001	2.53 (1.81–3.54)	< 0.001
Five portions of fruits and vegetables	1.08 (1.00–1.16)	0.044	1.34 (0.88–2.04)	0.174	1.42 (0.94–2.16)	0.099
Health screening						
General physical examination in past 3 y, n (%)	1.27 (1.18–1.36)	< 0.001	1.02 (0.71–1.47)	0.906	1.24 (0.85–1.82)	0.267
Cancer screening practice						
Any cancer screening practice^b^	1.61 (1.52–1.70)	< 0.001	0.18 (0.12–0.27)	< 0.001	0.50 (0.36–0.68)	< 0.001
Fecal occult blood test in past 2 y	2.53 (2.37–2.71)	< 0.001	1.31 (0.91–1.89)	0.149	3.26 (2.43–4.37)	< 0.001
Health-promoting activity in the past year						
Any health-promoting activity	1.14 (1.08–1.20)	< 0.001	1.19 (0.91–1.57)	0.206	2.41 (1.83–3.17)	< 0.001
Attend lectures	1.02 (0.96–1.08)	0.539	0.80 (0.56–1.15)	0.228	1.22 (0.90–1.66)	0.196
Participation in sports-related clubs	1.32 (1.24–1.40)	< 0.001	1.73 (1.29–2.33)	< 0.001	2.94 (2.24–3.85)	< 0.001
Use of gym or sport equipment	1.14 (1.04–1.25)	0.007	1.77 (1.17–2.69)	0.007	3.26 (2.33–4.57)	< 0.001
Participation in weight-control groups	1.51 (1.39–1.64)	< 0.001	0.82 (0.49–1.36)	0.439	2.36 (1.67–3.34)	< 0.001
Participation in recreational or service clubs	1.11 (1.01–1.22)	0.027	1.23 (0.77–1.98)	0.381	2.24 (1.54–3.25)	< 0.001

*aOR* adjusted Odds Ratio, *CI* confidence interval, *HPH* health-promoting hospital; *y* years.

^a^Odds ratio adjusted for participant characteristics (age, education level, marital status), health status (obese status, presence or absence of chronic disease), health behavior (smoking and drinking status), work unit, and hospital characteristics (accreditation level and ownership).

^b^Any cancer screening practice included pap smear test in the past 3 years, mammography in the past 2 years, and fecal occult blood test in the past 2 years in female nurses, but refereed to only fecal blood test in the past 2 years in male nurses.

**Table 4:** Adjusted odds ratio of health-related behaviors and screening practices associated with the interaction effect of health-promoting certification status of hospital and age (reference: younger than 40y and non-HPH group) (N = 26,011).

Outcomes	< 40y and HPH group aOR (95%CI)^a^	*p-*value	≥ 40y and Non-HPH group aOR (95%CI)^a^	*p-*value	≥ 40y and HPH group aOR (95%CI)^a^	*p-*value
Health behavior (at least one day during the past week)						
Exceeding 30 min of walking or equivalent physical activity	1.11 (1.05–1.18)	< 0.001	1.40 (1.24–1.58)	< 0.001	1.63 (1.47–1.82)	< 0.001
Five portions of fruits and vegetables	1.05 (0.98–1.14)	0.179	1.32 (1.10–1.58)	0.003	1.71 (1.45–2.03)	< 0.001
Health screening						
General physical examination in past 3 y, n (%)	1.27 (1.18–1.36)	< 0.001	2.23 (1.85–2.70)	< 0.001	2.86 (2.39–3.44)	< 0.001
Cancer screening practice						
Any cancer screening practice^b^	1.63 (1.53–1.73)	< 0.001	2.18 (1.91–2.50)	< 0.001	3.45 (3.04–3.91)	< 0.001
Pap smear in past 3 y (female nurses only)	1.19 (1.11–1.28)	< 0.001	1.59 (1.39–1.82)	< 0.001	2.24 (1.99–2.53)	< 0.001
Mammography in the past 2 y (female nurses only)	1.09 (0.97–1.23)	0.142	3.23 (2.75–3.79)	< 0.001	6.43 (5.61–7.36)	< 0.001
Fecal occult blood test in past 2 y	2.70 (2.50–2.91)	< 0.001	2.71 (2.37–3.09)	< 0.001	5.46 (4.88–6.11)	< 0.001
Health-promoting activity in the past year						
Any health-promoting activity	1.13 (1.07–1.19)	< 0.001	1.28 (1.15–1.43)	< 0.001	1.63 (1.48–1.80)	< 0.001
Attend lectures	1.01 (0.95–1.08)	0.710	1.59 (1.41–1.80)	< 0.001	1.73 (1.55–1.92)	< 0.001
Participation in sports-related clubs	1.29 (1.21–1.38)	< 0.001	0.82 (0.71–0.95)	0.007	1.25 (1.11–1.40)	< 0.001
Use of gym or sport equipment	1.06 (0.95–1.17)	0.292	1.36 (1.13–1.64)	0.001	2.10 (1.80–2.44)	< 0.001
Participation in weight-control groups	1.47 (1.34–1.61)	< 0.001	1.37 (1.16–1.63)	< 0.001	2.45 (2.14–2.79)	< 0.001
Participation in recreational or service clubs	1.05 (0.95–1.17)	0.362	1.55 (1.29–1.85)	< 0.001	2.16 (1.86–2.51)	< 0.001

*aOR* adjusted Odds Ratio, *CI* confidence interval, *HPH* health-promoting hospital, *y* years.

^a^Odds ratio adjusted for participant characteristics (sex, education level, marital status), health status (obese status, presence or absence of chronic disease), health behavior (smoking and drinking status), work unit, and hospital characteristics (accreditation level and ownership).

^b^Any cancer screening practice included pap smear test in the past 3 years, mammography in the past 2 years, and fecal occult blood test in the past 2 years in female nurses, but refereed to only fecal blood test in the past 2 years in male nurses.

**Table 5:** Adjusted odds ratio of health-related behaviors and screening practices associated with the interaction effect between health-promoting certification status of hospital and the presence or absence of chronic disease (reference: absence of chronic disease and non-HPH group) (N = 26,011).

Outcomes	Absence of chronic disease and HPH group aOR (95%CI)^a^	*p-*value	Presence of chronic disease and Non-HPH group aOR (95%CI)^a^	*p-*value	Presence of chronic disease and HPH group aOR (95%CI)^a^	*p-*value
Health behavior (at least one day during the past week)						
Exceeding 30 min of walking or equivalent physical activity	1.11 (1.04–1.18)	< 0.001	0.78 (0.72–0.86)	< 0.001	0.91 (0.84–0.99)	0.024
Five portions of fruits and vegetables	1.07 (0.98–1.16)	0.127	0.77 (0.69–0.87)	< 0.001	0.85 (0.76–0.95)	0.003
Health screening						
General physical examination in past 3 y, n (%)	1.30 (1.20–1.41)	< 0.001	1.07 (0.95–1.20)	0.251	1.26 (1.13–1.40)	< 0.001
Cancer screening practice						
Any cancer screening practice^b^	1.62 (1.52–1.73)	< 0.001	1.17 (1.06–1.29)	0.001	1.89 (1.73–2.06)	< 0.001
Pap smear in past 3 y (female nurses only)	1.22 (1.13–1.31)	< 0.001	1.19 (1.06–1.32)	0.002	1.48 (1.34–1.63)	< 0.001
Mammography in the past 2 y (female nurses only)	1.41 (1.25–1.57)	< 0.001	1.15 (0.98–1.35)	0.077	1.53 (1.34–1.76)	< 0.001
Fecal occult blood test in past 2 y	2.67 (2.46–2.89)	< 0.001	1.24 (1.10–1.39)	< 0.001	2.77 (2.52–3.05)	< 0.001
Health-promoting activity in the past year						
Any health-promoting activity	1.13 (1.06–1.19)	< 0.001	0.99 (0.90–1.07)	0.746	1.20 (1.11–1.29)	< 0.001
Attend lectures	1.02 (0.95–1.10)	0.528	0.99 (0.90–1.10)	0.859	1.03 (0.94–1.12)	0.591
Participation in sports-related clubs	1.29 (1.20–1.38)	< 0.001	0.92 (0.82–1.02)	0.110	1.32 (1.21–1.45)	< 0.001
Use of gym or sport equipment	1.16 (1.04–1.29)	0.009	0.99 (0.85–1.17)	0.942	1.15 (1.00–1.33)	0.044
Participation in weight-control groups	1.53 (1.39–1.69)	< 0.001	1.14 (0.98–1.31)	0.079	1.74 (1.54–1.96)	< 0.001
Participation in recreational or service clubs	1.14 (1.03–1.27)	0.016	0.97 (0.83–1.13)	0.682	1.05 (0.91–1.21)	0.504

*aOR* adjusted Odds Ratio, *CI* confidence interval, *HPH* health-promoting hospital, *y* years.

^a^Odds ratio adjusted for participant characteristics (age, sex, education level, marital status), health status (obese status), health behavior (smoking and drinking status), work unit, and hospital characteristics (accreditation level and ownership).

^b^Any cancer screening practice included pap smear test in the past 3 years, mammography in the past 2 years, and fecal occult blood test in the past 2 years in female nurses, but refereed to only fecal blood test in the past 2 years in male nurses.

We further stratified our study cohort by sex (Table 6). After covariate adjustment, the HPH effect was associated with an increased likelihood to perform physical activity and receive at least one general physical examination in the past 3 years in female nurses. Female nurses of HPH hospitals had significantly higher screening practices of pap smear in the past 3 years (39.8% vs 37.7%; older than 30 years, 57.2% vs 53.3%) and mammography examination in the past 2 years (10.2% vs 7.7%; older than 45 years, 47.5% vs 33.7%) than those of non-HPH hospitals. On the other hand, male nurses of HPH hospitals had a much higher chance of undergoing fecal occult blood test in the past 2 years (aOR 2.80 vs 2.53) and participating in hospital-based health-promoting activities in the past year (aOR 2.13 vs 1.14) than female nurses. Table 7 stratified nurses into two age groups (18–39 y, 40–65 y). In both age strata, nurses in HPH hospitals had a similar tendency as in the study cohort before stratification to perform physical activity (aOR 1.12–1.16 vs 1.12) and receive at least one general physical examination in the past 3 years (aOR 1.26–1.30 vs 1.27), which suggested that both health practices were independent of age. We also carried out a stratification analysis by chronic disease status (Table 8). Nurses in HPH hospitals had a similar tendency as in the study cohort before stratification to perform physical activity (aOR 1.11–1.16 vs 1.12) and participate in health-promoting activities (aOR 1.12–1.21 vs 1.15), which suggested that both health practices were affected by the HPH effect, and not by the presence or absence of chronic disease. On the contrary, nurses in the HPH hospitals received at least one general physical examination in the past 3 years (aOR 1.17 ~ 1.30) and fecal occult blood test in the past 2 years (aOR 2.19 ~ 2.70) irrespective of their chronic disease status.

**Table 6:** Adjusted odds ratio of health-related behaviors and screening practices associated with the health-promoting certification status of the hospital, stratified by sex (N = 26,011).

Outcomes	Sex									
	Female	(n = 25,550)		aOR (95% CI)^b^	*p*-value	Male	(n = 461)		aOR (95% CI)^b^	*p*-value
	HPH Group (56.9%) n (%)	Non-HPH Group (43.1%) n (%)	*p*-value^a^			HPH Group (51.6%) n (%)	Non-HPH Group (48.4%) n (%)	*p*-value^a^		
Health behavior (at least one day in the past week)										
Exceeding 30 min of walking or equivalent physical activity	9751 (67.1)	7065 (64.1)	< 0.001	1.12 (1.06–1.18)	< 0.001	194 (81.5)	175 (78.5)	0.415	1.37 (0.84–2.24)	0.212
Five portions of fruits and vegetables	12,540 (86.3)	9414 (85.4)	0.049	1.08 (1.00–1.16)	0.046	212 (89.1)	197 (88.3)	0.803	1.18 (0.63–2.21)	0.615
Health screening										
General physical examination in the past 3 y	12,520 (86.2)	9081 (82.4)	< 0.001	1.27 (1.18–1.36)	< 0.001	205 (86.1)	185 (83.0)	0.345	1.44 (0.82–2.54)	0.202
Cancer screening practice										
Any cancer screening practice^c^	8064 (55.5)	5242 (47.6)	< 0.001	1.61 (1.52–1.70)	< 0.001					
Pap smear in past 3 y (female nurses only)	5777 (39.8)	4153 (37.7)	< 0.001	1.22 (1.15–1.31)	< 0.001					
Pap smear in past 3 y (female nurses older than 30 y)	4693^d^ (57.2)	3464^d^ (53.3)	0.000	1.28 (1.18–1.39)	< 0.001					
Mammography in the past 2 y (female nurses only)	1480 (10.2)	851 (7.7)	< 0.001	1.38 (1.26–1.52)^f^	< 0.001					
Mammography in the past 2 y (female nurses older than 45 y)	548 (47.5)	250 (33.7)	0.000	1.90 (1.55–2.31)^e^	< 0.001					
Fecal occult blood test in past 2 y	4033 (27.8)	1587 (14.4)	< 0.001	2.53 (2.37–2.71)	< 0.001	74 (31.1)	38 (17.0)	< 0.001	2.80 (1.71–4.59)	< 0.001
Health-promoting activity in the past year										
Any health-promoting activity	6634 (45.7)	4733 (43.0)	< 0.001	1.14 (1.08–1.20)	< 0.001	153 (64.3)	105 (47.1)	< 0.001	2.13 (1.41–3.21)	< 0.001
Attend lectures	3361 (23.1)	2516 (22.8)	0.577	1.02 (0.96–1.08)	0.524	60 (25.2)	40 (17.9)	0.058	1.59 (0.97–2.59)	0.065
Participation in sports-related clubs	3545 (24.4)	2255 (20.5)	< 0.001	1.32 (1.24–1.40)	< 0.001	105 (44.1)	72 (32.3)	0.009	1.75 (1.15–2.65)	0.009
Use of gym or sport equipment	1203 (8.3)	819 (7.4)	0.013	1.14 (1.03–1.25)	0.008	50 (21.0)	28 (12.6)	0.016	1.83 (1.07–3.11)	0.026
Participation in weight-control groups	1931 (13.3)	979 (8.9)	< 0.001	1.51 (1.39–1.64)	< 0.001	45 (18.9)	17 (7.6)	< 0.001	3.71 (1.91–7.20)	< 0.001
Participation in recreational or service clubs	1191 (8.2)	836 (7.6)	0.074	1.11 (1.01–1.22)	0.026	37 (15.5)	21 (9.4)	0.047	1.65 (0.90–3.05)	0.107

*aOR* adjusted Odds Ratio, *HPH* health-promoting hospital, *y* years.

^a^Unadjusted *p*-value (χ^2^ test).

^b^Odds ratio adjusted for participant characteristics (age, education level, marital status), health status (obese status, presence or absence of chronic disease), health behavior (smoking and drinking status), work unit, and hospital characteristics (accreditation level and ownership).

^c^Any cancer screening practice included pap smear test in the past 3 years, mammography in the past 2 years, and fecal occult blood test in the past 2 years in female nurses, but refereed to only fecal blood test in the past 2 years in male nurses.

^d^Female [Non-HPH (n = 6499; 44.2%)/HPH (n = 8206; 55.8%)].

^e^Female [Non-HPH (n = 741; 39.1%)/HPH (n = 1154; 60.9%)].

^f^Odds ratio adjusted for all variables listed in footnote b, except for age status.

**Table 7:** Adjusted odds ratio of health-related behaviors and screening practices associated with the health-promoting certification status of the hospital, stratified by age (N = 26,011).

Outcomes	Age									
	18–39 y	(n = 22,048)		aOR (95% CI)^b^	*p*-value	40–65 y	(n = 3963)		aOR (95% CI)^b^	*p*-value
	HPH group (56.4%) n (%)	Non-HPH group (43.6%) n (%)	*p*-value^a^			HPH Group (59.1%) n (%)	Non-HPH Group (40.9%) n (%)	*p*-value^a^		
Health behavior (at least 1 day in the past week)										
Exceeding 30 min of walking or equivalent physical activity	8222 (66.2)	6096 (63.4)	< 0.001	1.12 (1.05–1.18)	< 0.001	1723 (73.6)	1144 (70.6)	0.038	1.16 (1.00–1.34)	0.043
Five portions of fruits and vegetables	10,591 (85.2)	8149 (84.7)	0.278	1.05 (0.97–1.13)	0.202	2161 (92.3)	1462 (90.2)	0.022	1.36 (1.08–1.71)	0.009
Health screening										
General physical examination in the past 3 y	10,528 (84.7)	7782 (80.9)	< 0.001	1.26 (1.18–1.36)	< 0.001	2197 (93.8)	1484 (91.5)	0.007	1.30 (1.01–1.67)	0.038
Cancer screening practice										
Any cancer screening practice^c^	6209 (50.0)	4043 (42.0)	< 0.001	1.62 (1.53–1.72)	< 0.001	1929 (82.4)	1237 (76.3)	< 0.001	1.57 (1.32–1.87)	< 0.001
Pap smear in past 3 y (female nurses only)	4103^d^ (33.6)	3095^d^ (32.8)	0.243	1.19 (1.11–1.27)^g^	< 0.001	1674^d^ (72.5)	1058^d^ (66.7)	< 0.001	1.47 (1.24–1.74)^g^	< 0.001
Pap smear in past 3 y (female nurses older than 30 y)	3019^e^ (51.2)	2406^e^ (49.0)	0.022	1.21 (1.11–1.33)^h^	< 0.001					
Mammography in the past 2 y (female nurses only)	700^d^ (5.7)	517^d^ (5.5)	0.437	1.09 (0.97–1.22)^g^	0.163	780^d^ (33.8)	334^d^ (21.0)	< 0.001	2.03 (1.74–2.36)^g^	< 0.001
Mammography in the past 2 y (female nurses older than 45 y)						548^f^ (47.5)	250^f^ (33.7)	< 0.001	1.90 (1.55–2.31)^h^	< 0.001
Fecal occult blood test in past 2 y	3119 (25.1)	1153 (12.0)	< 0.001	2.72 (2.52–2.93)	< 0.001	988 (42.2)	472 (29.1)	< 0.001	1.95 (1.69–2.24)	< 0.001
Health-promoting activity in the past year										
Any health-promoting activity	5496 (44.2)	4026 (41.8)	< 0.001	1.13 (1.07–1.19)	< 0.001	1291 (55.1)	812 (50.1)	0.002	1.28 (1.12–1.46)	< 0.001
Attend lectures	2607 (21.0)	2015 (20.9)	0.950	1.01 (0.94–1.08)	0.857	814 (34.8)	541 (33.4)	0.367	1.11 (0.97–1.28)	0.132
Participation in sports-related clubs	3116 (25.1)	2047 (21.3)	< 0.001	1.30 (1.22–1.39)	< 0.001	534 (22.8)	280 (17.3)	< 0.001	1.49 (1.26–1.76)	< 0.001
Use of gym or sport equipment	899 (7.2)	676 (7.0)	0.552	1.06 (0.95–1.18)	0.272	354 (15.1)	171 (10.5)	< 0.001	1.54 (1.26–1.88)	< 0.001
Participation in weight-control groups	1459 (11.7)	782 (8.1)	< 0.001	1.46 (1.33–1.60)	< 0.001	517 (22.1)	214 (13.2)	< 0.001	1.84 (1.54–2.21)	< 0.001
Participation in recreational or service clubs	873 (7.0)	667 (6.9)	0.790	1.05 (0.94–1.17)	0.362	355 (15.2)	190 (11.7)	0.002	1.40 (1.15–1.70)	< 0.001

*aOR* adjusted Odds Ratio, *HPH* hospital promoting hospital, *y* years.

^a^Unadjusted *P*-value (χ^2^ test).

^b^Odds ratio adjusted for patient characteristics (sex, education level, marital status, body mass index, presence or absence of chronic disease, smoking and drinking status, hospital units) and hospital characteristics (accreditation level and ownership).

^c^Any cancer screening practice included pap smear test in the past 3 years, mammography in the past 2 years, and fecal occult blood test in the past 2 years in female nurses, but refereed to only fecal blood test in the past 2 years in male nurses.

^d^Female Age (18–39 y) [Non-HPH (n = 9432; 43.6%)/HPH (n = 12,223; 56.4%)]; Female Age (40–65 y) [Non-HPH (n = 1587; 40.7%)/HPH (n = 2308; 59.3%)].

^e^Female Age (30–39 y) [Non-HPH (n = 4912; 45.4%)/HPH (n = 5898; 54.6%)].

^f^Female Age (45–65 y) [Non-HPH (n = 741; 39.1%)/HPH (n = 1154; 60.9%)].

^g^Odds ratio adjusted for all variables listed in footnote b, except for the sex status.

^h^Odds ratio adjusted for all variables listed in footnote b, except for the sex and age status.

**Table 8:** Adjusted odds ratio of health-related behaviors and screening practices associated with the health-promoting certification status of the hospital, stratified by the presence or absence of chronic disease (N = 26,011).

Outcomes	Chronic disease status									
	0	(n = 18,921)		aOR (95% CI)^b^	*p*-value	≧1	(n = 7090)		aOR (95% CI)^b^	*p*-value
	HPH Group (56.4%) n (%)	Non-HPH Group (43.6%) n (%)	*p*-value^a^			HPH Group (57.8%) n (%)	Non-HPH Group (42.2%) n (%)	*p*-value^a^		
Health behavior (at least one day in the past week)										
Exceeding 30 min of walking or equivalent physical activity	7276 (68.2)	5408 (65.6)	< 0.001	1.11 (1.04–1.18)	< 0.001	2669 (65.1)	1832 (61.2)	< 0.001	1.16 (1.05–1.28)	0.005
Five portions of fruits and vegetables	9263 (86.8)	7104 (86.1)	0.165	1.07 (0.98–1.17)	0.115	3489 (85.1)	2507 (83.8)	0.120	1.09 (0.96–1.25)	0.181
Health screening										
General physical examination in the past 3 y	9173 (86.0)	6751 (81.8)	< 0.001	1.30 (1.20–1.41)	< 0.001	3552 (86.7)	2515 (84.1)	0.002	1.17 (1.02–1.34)	0.022
Cancer screening practice										
Any cancer screening practice^c^	5679 (53.2)	3747 (45.4)	< 0.001	1.62 (1.52–1.74)	< 0.001	2459 (60.0)	1533 (51.2)	< 0.001	1.59 (1.43–1.77)	< 0.001
Pap smear in past 3 y (female nurses only)	3955^d^ (37.6)	2929^d^ (36.1)	0.038	1.21 (1.12–1.31)^g^	< 0.001	1822^d^ (45.4)	1224^d^ (42.1)	0.006	1.25 (1.10–1.41)^g^	< 0.001
Pap smear in past 3 y (female nurses older than 30 y)	3105^e^ (56.4)	2395^e^ (52.7)	< 0.001	1.27 (1.15–1.40)^h^	< 0.001	1588^e^ (58.8)	1069^e^ (54.7)	0.006	1.29 (1.12–1.49)^h^	< 0.001
Mammography in the past 2 y (female nurses only)	964^d^ (9.2)	568^d^ (7.0)	< 0.001	1.41 (1.26–1.58)^g^	< 0.001	516^d^ (12.9)	283^d^ (9.7)	< 0.001	1.30 (1.10–1.54)^g^	0.002
Mammography in the past 2 y (female nurses older than 45 y)	294^f^ (49.0)	132^f^ (30.9)	< 0.001	2.37 (1.80–3.12)^h^	< 0.001	254^f^ (45.8)	118^f^ (37.6)	0.018	1.49 (1.10–2.00)^h^	0.009
Fecal occult blood test in past 2 y	2871 (26.9)	1110 (13.5)	< 0.001	2.70 (2.49–2.92)	< 0.001	1236 (30.2)	515 (17.2)	< 0.001	2.19 (1.94–2.47)	< 0.001
Health-promoting activity in the past year										
Any health-promoting activity	4806 (45.0)	3527 (42.8)	0.002	1.12 (1.06–1.19)	< 0.001	1981 (48.3)	1311 (43.8)	< 0.001	1.21 (1.10–1.34)	< 0.001
Attend lectures	2419 (22.7)	1849 (22.4)	0.675	1.02 (0.95–1.09)	0.618	1002 (24.5)	707 (23.6)	0.425	1.04 (0.92–1.16)	0.540
Participation in sports-related clubs	2618 (24.5)	1739 (21.1)	< 0.001	1.29 (1.20–1.38)	< 0.001	1032 (25.2)	588 (19.7)	< 0.001	1.43 (1.27–1.61)	< 0.001
Use of gym or sport equipment	883 (8.3)	608 (7.4)	0.022	1.15 (1.03–1.29)	0.011	370 (9.0)	239 (8.0)	0.122	1.16 (0.98–1.38)	0.089
Participation in weight-control groups	1330 (12.5)	682 (8.3)	< 0.001	1.52 (1.38–1.68)	< 0.001	646 (15.8)	314 (10.5)	< 0.001	1.55 (1.33–1.79)	< 0.001
Participation in recreational or service clubs	882 (8.3)	619 (7.5)	0.054	1.15 (1.03–1.28)	0.014	346 (8.4)	238 (8.0)	0.460	1.07 (0.89–1.27)	0.473

*aOR* adjusted Odds Ratio, *HPH* health-promoting hospital, *y* years.

^a^Unadjusted *P*-value (χ^2^ test).

^b^Odds ratio adjusted for participant characteristics (age, sex, education level, marital status), health status (obese status), health behavior (smoking and drinking status), work unit, and hospital characteristics (accreditation level and ownership).

^c^Any cancer screening practice included pap smear test in the past 3 years, mammography in the past 2 years, and fecal occult blood test in the past 2 years in female nurses, but refereed to only fecal blood test in the past 2 years in male nurses.

^d^Female Chronic disease status 0 [Non-HPH (n = 8111; 43.5%)/HPH (n = 10,522; 56.5%)]; Female Chronic disease status 1 [Non-HPH (n = 2908; 42.0%)/HPH (n = 4009; 58.0%)].

^e^Female Chronic disease status 0 [Non-HPH (n = 4545; 45.2%)/HPH (n = 5504; 54.8%)]; Female Chronic disease status 1 [Non-HPH (n = 1954; 42.0%)/HPH (n = 2702; 58.0%)].

^f^Female Chronic disease status 0 [Non-HPH (n = 427; 41.6%)/HPH (n = 600; 58.4%)]; Female Chronic disease status 1 [Non-HPH (n = 314; 36.2%)/HPH (n = 554; 63.8%)].

^g^Odds ratio adjusted for all variables listed in footnote b, except for the sex status.

^h^Odds ratio adjusted for all variables listed in footnote b, except for the sex and age status.

## Discussion

This study demonstrated that nurses in HPH hospitals were more likely to have better health practices, including health behavior (physical activity and five portions of fruits and vegetables), health screening, and health-promoting activity provided by hospitals when compared to those in non-HPH hospitals in Taiwan. In addition, the interaction between HPH status and individual differences (sex/age/chronic disease) was significant, meaning that associations between HPH status and health practices differed in those with different conditions of sex/age/chronic disease. Finally, our study suggested that nurses who worked in HPH hospitals were more likely to have better health practices, regardless of gender, age, or chronic disease.

In comparison to the general population or other hospital staff, nurses seemingly displayed poorer health-related behaviors and lifestyles^3,19,21,22^. The poorer health-related behavior and lifestyle of nursing professionals could be partially explained by gender differences^23^. In the United States, 47.9 percent of adults participated in moderate-intensity aerobic physical activity of at least 150 min per week or 600 MET-minutes per week in 2020^24^. About 52.7% Taiwanese population achieves 600 MET minutes per week, with 61% in males and 44.8% in females^25^. However, we found that only 12.27% of Taiwanese nurses do 30-min brisk walking of at least 5 days per week, with 19.5% in males and 12.1% in females. Despite the male nurses are better at physical activity than female nurses, the rate did not meet the recommendation and was obviously lower than the Taiwanese general population. Besides physical activity, our research also indicated that male nurses are more likely to participate in sports-related clubs and use gym or sports equipment when compared to female nurses. Interestingly, both male and female nurses who work in HPH tend to have better health practices than those in non-HPH settings. Considering that nurses represent the largest occupational group in hospitals, there is a pressing need to enhance health-promoting initiatives in hospitals and improve the health-promoting practices of nurses, especially female nurses. To encourage participation, female-specific sports-related equipment and clubs could be provided for female nurses.

HPH status and age/chronic disease status were found to be considered simultaneously to predict health practices among nurses^3,19^. Nurses who were aged 40 and above in HPH were more likely to engage in health behaviors, participate in health screening, and join health-promoting activities facilitated by hospitals than those who were aged below 40 years in non-HPH settings. Additionally, nurses who had a chronic disease in HPH were more likely to participate in cancer screening and join health-promoting activities provided by hospitals when compared to those without chronic disease in non-HPH settings. Given the relationship between increasing age and the prevalence of chronic disease, the positive impact of HPH status on health practices appeared to be similar when stratified by age or baseline chronic disease. Our result further indicated that the interaction between age and baseline chronic disease was associated with health practices among nurses. Nurses who were 40 years of age or older, regardless of their chronic disease status, exhibited a higher likelihood of engaging in healthy practices. It is noteworthy that nurses who were less than 40 years of age and had a chronic disease demonstrated a higher likelihood of engaging in any cancer screening practice, while this group of nurses exhibited a lower likelihood of engaging in certain health behaviors such as exceeding 30 min of walking and consuming five portions of fruits and vegetables a day (see Supplementary Table S1). It implies that younger nurses tend to seek health checks only when they experience a health problem. These findings highlight the importance of considering individual differences, such as age and baseline chronic disease, when promoting health practices among nurses. Healthcare organizations and policymakers should consider tailoring interventions based on individual characteristics to encourage and support health practices among healthcare professionals.

Research suggests that nurses who exhibit healthy behaviors themselves are more likely to provide information about such behaviors to their patients^9–13^. In our study, most nurses reported having undergone a general physical examination within the past three years and consuming at least five portions of fruits and vegetables on at least one day during the past week. However, the rates of participation in hospital-based health-promoting activities during the past year were low (7.5–24.7%) among nurses, regardless of HPH status. Overall, male nurses, elder nurses, and nurses with chronic diseases were more likely to participate in hospital-based clubs or groups related to sports and weight control, particularly in HPH. These findings can inform policymakers in designing appropriate activities for male nurses, elder nurses, and nurses with chronic diseases, while also increasing incentives for female nurses, younger nurses, and nurses without chronic diseases to participate in such activities and promote healthy habits for themselves and their patients.

### Limitation

The cross-sectional study design is limited in establishing a causal relationship. The data were self-reported, which may not exclude a recall bias and response bias. We have no information on nurses who refused to participate in this study, which may have a selection bias. Only 461 male nurses were recruited in this study (1.77%). Hence, our results were probably underpowered to detect the small differences in health-related behaviors between male nurses who worked in HPH or in non-HPH. The questions regarding the physical activities and consumption of fruit and vegetables were not measured using participants' actual habits.

## Conclusions

This study suggests the effectiveness of implementing health promotion hospital programs on the health practices of full-time nursing staff in hospitals. To promote the health practice of nurses, their workplace could participate HPH network and provide policies and a supportive environment regarding health promotion to improve nurses’ health. To increase health-promoting activities provided by hospitals and health behavior in female nurses, female-specific lectures, equipment, and clubs could be provided to increase their motions to participation. Male nurses, elder nurses, and nurses with chronic diseases are more likely to have health-related behaviors. Appropriate activities could be designed for male nurses, elder nurses, and nurses with chronic disease and incentives could be increased for female nurses, younger nurses, and nurses without chronic disease to further drive them and their patients to have healthy habits. Future studies can apply a longitudinal design to further examine the effect of HPH status on changes in health practice over time among nursing staff.

## Supplementary Information

Supplementary Figure S1.

Supplementary Table S1.

Supplementary Legends.

## Data Availability

The data that support the findings of this study are available from Health Promotion Administration, Taiwan (ROC) but restrictions apply to the availability of these data, which were used under license for the current study, and so are not publicly available. Data are however available from the corresponding author Li-Yin Chien upon reasonable request if permission is granted by the Health Promotion Administration, Taiwan (R.O.C.).
