# Supplementary material for: The effect of hospital-based health promotion on the health practices of full-time hospital nurses: a cross-sectional study
Source: Sci Rep. 2023 Jun 16;13:9763. doi: 10.1038/s41598-023-36873-z (PMC10275936; doi:10.1038/s41598-023-36873-z)
Supplement: Supplementary file 2 — Supplementary Table S1. [file 41598_2023_36873_MOESM2_ESM.docx]

Table S1. Adjusted odds ratio of health-related behaviors and screening practices associated with the interaction effect between chronic disease and age (reference: absence of chronic disease and younger than 40y) (N = 26,011).

| **Outcomes** | **Presence of chronic disease and younger than 40y**  **aOR (95%CI)^a^** | ***p-*value** | **Absence of**  **chronic disease and older than 40y**  **aOR (95%CI)^a^** | ***p-*value** | **Presence of chronic disease and older than 40y**  **aOR (95%CI)^a^** | ***p-*value** |
| --- | --- | --- | --- | --- | --- | --- |
| **Health behavior (at least one day during the past week)** | |  |  |  |  |  |
| **Exceeding 30 minutes of walking or equivalent physical activity** | 0.78 (0.73-0.84) | <0.001 | 1.35 (1.22-1.50) | <0.001 | 1.25 (1.11-1.40) | <0.001 |
| **Five portions of fruits and vegetables** | 0.79 (0.72-0.85) | <0.001 | 1.48 (1.26-1.75) | <0.001 | 1.17 (0.97-1.40) | 0.093 |
| **Health screening** |  |  |  |  |  |  |
| **General physical examination in past 3 y, n (%)** | 0.99 (0.91-1.08) | 0.822 | 2.06 (1.75-2.43) | <0.001 | 2.59 (2.09-3.20) | <0.001 |
| **Cancer screening practice** |  |  |  |  |  |  |
| **Any cancer screening practice^b^** | 1.17 (1.10-1.26) | <0.001 | 2.19 (1.95-2.46) | <0.001 | 2.46 (2.14-2.83) | <0.001 |
| **Pap smear in past 3 y (female nurses only)** | 1.22 (1.13-1.32) | <0.001 | 1.80 (1.61-2.01) | <0.001 | 2.04 (1.78-2.34) | <0.001 |
| **Mammography in the past 2 y (female nurses only)** | 0.98 (0.86-1.13) | 0.809 | 4.27 (3.76-4.84) | <0.001 | 5.49 (4.79-6.30) | <0.001 |
| **Fecal occult blood test in past 2 y** | 1.07 (0.99-1.16) | 0.074 | 2.17 (1.96-2.41) | <0.001 | 2.56 (2.27-2.88) | <0.001 |
| **Health-promoting activity in the past year** |  |  |  |  |  |  |
| **Any health-promoting activity** | 1.02 (0.96-1.09) | 0.448 | 1.37 (1.25-1.50) | <0.001 | 1.44 (1.29-1.60) | <0.001 |
| **Attend lectures** | 0.99 (0.92-1.07) | 0.777 | 1.63 (1.48-1.81) | <0.001 | 1.68 (1.49-1.88) | <0.001 |
| **Participation in sports-related clubs** | 0.98 (0.91-1.05) | 0.548 | 0.90 (0.80-1.01) | 0.075 | 0.90 (0.79-1.03) | 0.133 |
| **Use of gym or sport equipment** | 0.96 (0.85-1.08) | 0.526 | 1.65 (1.42-1.91) | <0.001 | 1.79 (1.52-2.12) | <0.001 |
| **Participation in weight-control groups** | 1.11 (1.01-1.23) | 0.036 | 1.52 (1.33-1.73) | <0.001 | 1.82 (1.58-2.10) | <0.001 |
| **Participation in recreational or service clubs** | 0.91 (0.80-1.03) | 0.121 | 1.77 (1.53-2.05) | <0.001 | 1.80 (1.52-2.12) | <0.001 |
