# Supplementary material for: The effect of hospital-based health promotion on the health practices of full-time hospital nurses: a cross-sectional study
Source: Sci Rep. 2023 Jun 16;13:9763. doi: 10.1038/s41598-023-36873-z (PMC10275936; doi:10.1038/s41598-023-36873-z)
Supplement: Supplementary file 3 — Supplementary Legends. [file 41598_2023_36873_MOESM3_ESM.doc]

**TABLE LEGEND**

Table S1. Adjusted odds ratio of health-related behaviors and screening practices associated with the interaction effect between chronic disease and age (reference: absence of chronic disease and younger than 40y) (N = 26,011). Abbreviations: aOR, adjusted Odds Ratio; CI, confidence interval; HPH, health-promoting hospital; y, years. ^a^Odds ratio adjusted for participant characteristics (sex, HPH, education level, marital status), health status (obese status), health behavior (smoking and drinking status), work unit, and hospital characteristics (accreditation level and ownership). ^b^Any cancer screening practice included pap smear test in the past 3 years, mammography in the past 2 years, and fecal occult blood test in the past 2 years in female nurses, but referred to only fecal blood test in the past 2 years in male nurses.

**FIGURE LEGEND**

Figure S1. Flowchart of participants.
